# Integrins as Key Mediators of Metastasis

**DOI:** 10.3390/ijms26030904

**Published:** 2025-01-22

**Authors:** Daniel Cáceres-Calle, Irene Torre-Cea, Laura Marcos-Zazo, Iván Carrera-Aguado, Elena Guerra-Paes, Patricia Berlana-Galán, José M. Muñoz-Félix, Fernando Sánchez-Juanes

**Affiliations:** 1 Departamento de Bioquímica y Biología Molecular, Universidad de Salamanca, 37007 Salamanca, Spain; danicc@usal.es (D.C.-C.); irenedltorrecea@usal.es (I.T.-C.); lauramarcos@usal.es (L.M.-Z.); icasdp@usal.es (I.C.-A.); elegpaes98@usal.es (E.G.-P.); berlanagalan@usal.es (P.B.-G.); 2 Instituto de Investigación Biomédica de Salamanca (IBSAL), 37007 Salamanca, Spain

**Keywords:** integrins, metastasis, extracellular matrix, tumor blood vessels

## Abstract

Metastasis is currently becoming a major clinical concern, due to its potential to cause therapeutic resistance. Its development involves a series of phases that describe the metastatic cascade: preparation of the pre-metastatic niche, epithelial–mesenchymal transition, dissemination, latency and colonization of the new tissue. In the last few years, new therapeutic targets, such as integrins, are arising to face this disease. Integrins are transmembrane proteins found in every cell that have a key role in the metastatic cascade. They intervene in adhesion and intracellular signaling dependent on the extracellular matrix and cytokines found in the microenvironment. In this case, integrins can initiate the epithelial–mesenchymal transition, guide the formation of the pre-metastatic niche and increase tumor migration and survival. Integrins also take part in the tumor vascularization process necessary to sustain metastasis. This fact emphasizes the importance of inhibitory therapies capable of interfering with the function of integrins in metastasis.

## 1. The Metastatic Process: From Pre-Metastatic Niche to Distant Organ Colonization

Cancer is a pathology of worldwide health importance due to the large number of people affected, knowing that currently one in five people will develop this disease [1,2]. The most recent studies indicate that, globally, it has an incidence of 20 million people, accompanied by a mortality of 9.7 million [1,2]. The most common malignant neoplasms worldwide are lung, breast, colorectal, prostate and stomach cancer [1,2]. A 77% increase in new cases is expected by 2050, reflecting growth and aging in society [1]. More advances are being made to personalize diagnosis and treatment, depending on the type of tumor and the clinical resistance that may appear. However, we must bear in mind that within these statistics, metastasis is one of the lethal manifestations of this disease [3].

Metastasis is a systemic disease [4]. It consists in the dissemination of cells from a primary tumor to a distal organ through the bloodstream or lymphatic system [3,5]. It is a significant clinical complication for both detection and treatment, due to the high probability of relapses, intratumoral phenotypic plasticity and acquired resistance to current therapies [4,6,7].

The lethality of these types of neoplasms is due to the fact that highly malignant cell subtypes are selected during the metastatic process [3,5,8]. Cells that colonize secondary sites have acquired the appropriate characteristics to survive after the previous steps of the metastatic cascade [7,9,10]. This is because of the colonization step, which is the limiting phase of metastasis [7].

Even though all organs and tissues can be destinations of these cells, some organs seem to be more prone to be colonized. Moreover, it is currently known that the primary tumor prepares the new distant tissue susceptible to developing a secondary tumor [3,11,12]. This is called organic tropism, and it depends on the physical location of the target organ and the microenvironment [3,13]. For example, breast cancer usually metastasizes to bone, lung and brain tissues [8]. The primary tumor secretes exosomes with pro-tumor signals to the target organ, initiating a remodeling of the extracellular matrix (ECM) and the cell populations that will create a favorable tumor microenvironment (TME) [11,12,13]. It is a genetic and phenotypic process in different cell types that involves different intracellular signals to form the oncogenic stroma [14,15,16,17,18].

While colonization is a crucial phase, metastasis is a set of complex stages that overlap in time, which can be classified into dissemination, latency and colonization [3,9].

The formation of pre-metastatic niches (PMNs) in distant sites is essential to attract tumor cells to the target tissue [8,19]. They are generated through the arrival of extracellular vesicles (EVs) carrying signals such as S100, tumor necrosis factor alpha (TNF-α) and transforming growth factor beta (TGF-β) to modify vascular permeability. The ECM and cancer cells reprogram resident immune cells, generating a pro-inflammatory environment promoted by cytokines like interleukin 6 (IL-6) and interleukin 8 (IL-8) [8,13,20]. Cancer-associated fibroblasts (CAFs) also induce a pro-fibrotic state by releasing numerous regulatory factors that affect intercellular interaction and ECM remodeling [19,21]. CAFs appear to assist in integrin β1-mediated EV transport into the PMN of lung metastasis [22]. They also display potentially targetable signaling for diagnosis and treatment, such as the ZNF281-CCL2/CCL5 downstream pathway in breast cancer [19] or the YAP1/GROα/CXCRs pathway induced in fibroblasts to convert them into CAFs in ovarian cancer [23]. On the contrary, TRAIL upregulation appears to decrease PMN effectiveness for potential colonization [24].

### The Metastatic Cascade

**Figure 1:**
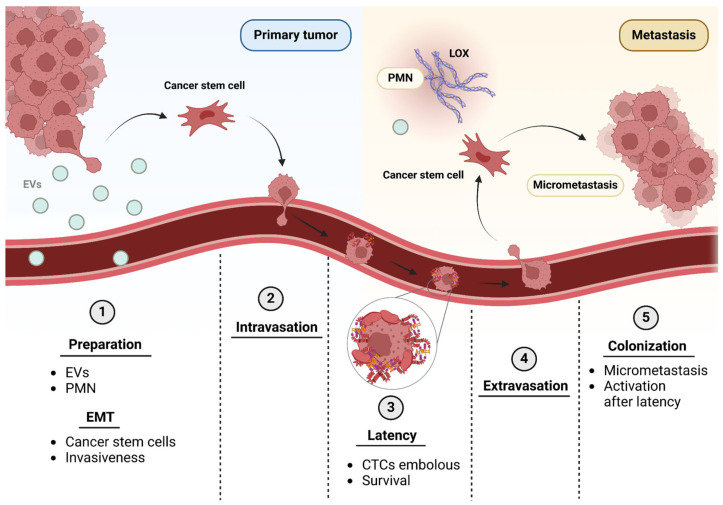
**The metastatic cascade.** For development of metastasis, cells from the primary tumor prepare the tissue of distant sites by secreting extracellular vesicles (EVs) into the bloodstream. (**1**) This induces the PMN with matrix deposition by stimulating lysyl oxidases (LOX) in the target tissue. In turn, the EMT program begins where tumor cells with an epithelial phenotype become mesenchymal or cancer stem cells, acquiring greater invasiveness among other highly malignant characteristics. (**2**) Cells that undergo EMT tend to intravasate through the basal membrane and the endothelium into the bloodstream. (**3**) Once they are in the bloodstream, circulating tumor cells (CTCs) are in a latent state or in proliferative quiescence and form an embolus with FN and platelets that helps their survival during dissemination. (**4**) When these cells reach the target tissue, they extravasate, crossing back through the endothelium and the basal membrane. (**5**) If a favorable PMN is found in this tissue, colonization will begin, losing the state of latency towards a proliferative state, forming at first a clinically undetectable micrometastasis, which can evolve into a properly known metastasis. Created with BioRender.com.

Before the dissemination of tumor cells from a primary tumor takes place, a series of genetic and phenotypic modifications must occur in them. In order to metastasize, it is essential that tumor cells acquire an aggressive phenotype, undergoing numerous events such as the epithelial–mesenchymal transition (EMT) program [3,5,9,25,26]. It consists of the transdifferentiation of an epithelial phenotype to a mesenchymal one. Different studies and computational models support that this phenotype may be not completely acquired but that hybrid cells coexist between epithelial and mesenchymal states in the microenvironment. This partial or hybrid epithelial/mesenchymal phenotype has positive markers for both cell types, such as CD24+/CD44+, and shows a pluripotency typical of cancer stem cells (CSC) involved in tumor initiation [27]. In this process, molecules involved in epithelial polarity or adhesion such as ZO-1 or E-cadherin are downregulated, while mesenchymal markers such as α-SMA or fibronectin (FN) are upregulated, changing from an epithelial phenotype to a mesenchymal one, which gives them a greater capacity for migration, invasiveness and survival [26,27,28] (Figure 1). In recent years, hybrid EMT has also been linked to acquired resistance to therapies, for example, to epithelial growth factor receptor (EGFR) and PI3K/Akt inhibitors, and the AXL tyrosine kinase receptor was found to be a common therapeutic target for several cancer types [15,16,27,29]. This is also related to the phenotype found in metastases from breast cancer, as is the case of metastases in lymph nodes where cells with transcriptional states with a mesenchymal state are usually found [30].

Some of these cells that undergo EMT reach a blood or lymphatic vessel favored by the remodeling of the tumor ECM. Here begins the metastatic dissemination phase that can be divided into the stages of intravasation, survival in transport and extravasation (Figure 1). First, the primary tumor cell intravases through the endothelium and reaches the blood or lymphatic stream, where they are called circulating tumor cells (CTCs) [31,32]. For survival in the vascular lumen, CTCs surround themselves with platelets, fibrin and FN, forming an embolus that protects them from hemodynamic forces and immune responses [32,33]. Furthermore, CTCs are in a dormant state [32,33]. It has been experimentally observed that polyclonal groups of CTCs survive transport better than individual tumor cells [34]. The next step is extravasation, for which the embolus is stopped in the vessel, aided by FN, membrane receptors and physical parameters [32,33]. Upon extravasation, they cross the endothelium again with the help of transmembrane proteins such as integrin αvβ3 and proteases [32]. After dissemination, selected tumor clones are competent to survive all the unfavorable conditions of the metastatic journey (Figure 1).

The latency phase is a state of proliferative quiescence that begins in CTCs and continues after they have extravasated [33]. Once the metastatic cells reach the new tissue, they can enter a partial state of latency or dormancy, finding a balance between apoptosis and proliferation: this is how a micrometastasis is maintained, which is often clinically undetectable [28,32,33]. After an indefinite period of time, the colonization stage of the new tissue follows. The cells completely leave the latency state, begin to proliferate and migrate, invading the new tissue, forming a metastatic tumor [33] (Figure 1).

## 2. ECM in Healthy Tissues and Its Involvement in Metastasis

The ECM is a dynamic 3D structure formed by macromolecules that provides support and regulates biological functions of the cancer cells where the cell populations of a microenvironment tissue develop. It provides support to cells, organizes tissue zones, allows cell adhesion and mobility and intervenes in intracellular communication [35,36,37].

The ECM can be divided into pericellular ECM and interstitial ECM [35,37]. The pericellular matrix is an organized network that facilitates cell attachment by binding with integrins, discoidin domain receptors (DDRs) and proteoglycans [37]. The interstitial matrix provides tissue integrity that divides and supports different tissues [37]. Similarly, basement membranes (BMs) are fibrous structures made up mostly of type IV collagen and laminin, which are located beneath the epithelium and act as a support and barrier [38].

The components of the ECM are synthesized by the stromal cells themselves, such as fibroblasts [39]. This is possible thanks to the matrisome, a set of genes that contain the information necessary for the synthesis of ECM and the proteins associated with it [40]. Moreover, some of these genes encode for lysyl oxidase proteins (LOX), which are involved in matrix crosslinking [37].

The ECM is mainly made up of proteins such as collagens, elastin, lamellae, proteoglycans and glycosaminoglycans, as well as the cellular receptors that interact with them, such as CD44 and integrins [37]. Collagens have a characteristic triple helix morphology and are the main components of the ECM. There are different types, although the most abundant in general are type I, II and III [35,37]. However, each tissue has its characteristic collagen seal. For example, types X, XI, XII, XIV, XX, XXIV and XXVII are mainly present in connective tissues such as tendons and cartilage [37,41]. On the other hand, FN is also important in the composition of the ECM; it forms supramolecular complexes that help regulate the mechanical properties of the tissue and contain cytokines involved in cellular regulation. Other components are laminins and glycoproteins, which are an essential part of BMs; and proteoglycans that may contain, to a greater or lesser extent, hyaluronic acid involved in tissue homeostasis [35,37]. Proteoglycans, such as heparan, heparin or chondroitin sulfate, provide mechanical resistance to the tissue, in addition to trapping growth factors and water [42].

Under physiological conditions, a balance between ECM synthesis and degradation, known as remodeling, is essential for tissue homeostasis. Proteases and glycoproteases, such as metalloproteinases (MMPs), play a key role in ECM degradation [39]. Disruption of this balance can lead to pathological conditions. Diseases like cancer can alter ECM remodeling to facilitate their growth and dissemination.

The ECM is a key element of metastatic development. Its components and the cells that reside in them can facilitate or slow down tumor colonization [43,44]. Furthermore, as we mentioned previously, the ECM begins to be modified in favor of cancer from the formation of PMNs; for example, an overexpression of LOX induced by EVs associated with primary breast cancer has been observed [45,46]. Throughout the entire metastatic process, matrix stiffness is increased by depositing FN and collagen, facilitating the advancement of cancer [44,47]. This is an important step because colonizing cells need support to migrate along the TME [48,49,50]. It must be taken into account that tumor cells also modulate the remodeling of the ECM, creating a highly organized structure and regulating the level of rigidity, since high deposition can prevent cell mobility [49,50,51,52,53]. Recent studies indicate that CAFs replace the healthy tissue matrix with a tumoral matrix, depositing type XII collagen as a critical component in metastatic adenocarcinoma [54].

In the metastatic dissemination phase, involvement with the ECM is crucial because tumor cells have to break through, traverse and surround themselves with fibers of the endothelial BM [55]. In tumor colonization and invasion, cells pass through collagen fibers through invadopodia that contain MMPs. In this process, intracellular signaling is transmitted in part by adhesion to the ECM and the signals found therein, such as TGF-β, HGF (hepatocyte growth factor) and growth factors derived from CAFs such as SDF1/CXCL12 [51].

At this point, an important link between the ECM and tumor cells are integrins, ECM receptors that participate in cell adhesion, migration and communication. Therefore, below, we delve deeper into integrins and their involvement in the metastatic process.

## 3. Integrins

**Figure 2:**
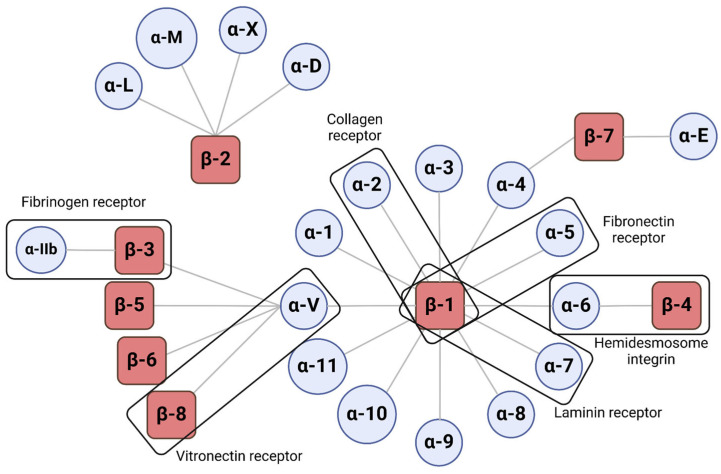
**Integrin αβ heterodimer ligands.** The α- and β-subunits interact as depicted above, forming different integrins with different ligands. Some indicated examples are integrins of great interest, such as α2β1, which recognizes collagen fibers as ligands, or α5β1, an FN receptor. Created with BioRender.com.

Integrins are heterodimeric transmembrane receptors containing large extracellular domains and mostly short cytoplasmic domains [56]. Integrins bind extracellular ligands present in ECM, each one with unique ligand and signaling faculties [57]. The structure of integrins consists of an α-subunit and a β-subunit that interact with each other to form the heterodimeric receptor. These interactions will dictate its intracellular signaling pathway, the activation kinetics and the stability of its different forms. Currently, 18 α-subunit and 8 β-subunit subtypes have been described, making it possible to form 24 heterodimers in mammals. (Figure 2) [56,58].

### 3.1. Structure and Function

**Figure 3:**
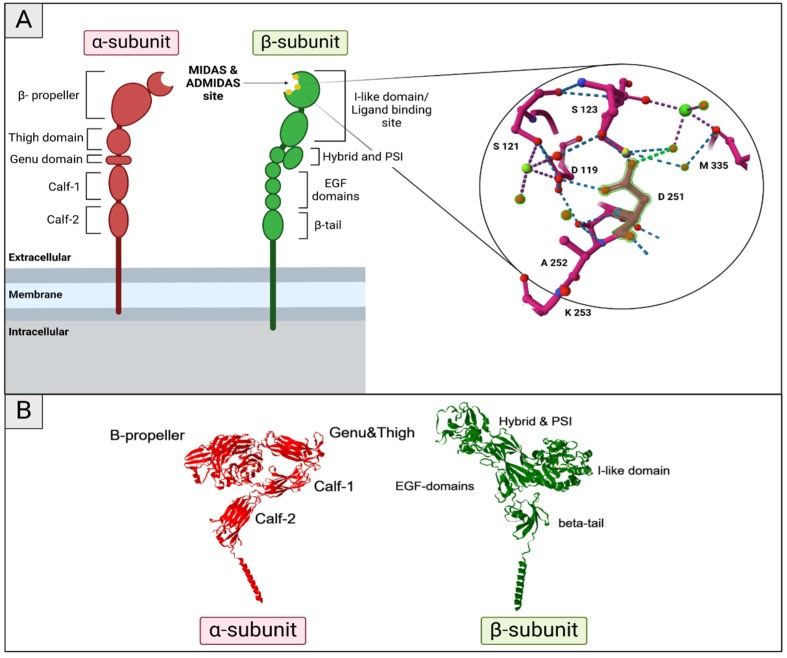
**Structure of integrins.** (**A**) Schematic depiction of integrin structure with a close look on the MIDAS and ADMIDAS sites, PDB: 8T2V [65]. The upper leg of the α-subunit consists of a thigh and genu (knee-like) domain, and the lower leg is formed by the calf-1 and calf-2 domains. The C-terminal part of the subunit is organized in a β-propeller structure. β-subunit topology is more complex. The bottom is formed by a β-tail followed by up to four epithelial growth factor (EGF)-like domains. After these, a hybrid domain is inserted in the plexin/semaphorin/integrin (PSI) domain. The remainder C-terminal is the β-subunit ‘inserted’ (I) domain that is inserted in the hybrid domain, which forms the upper portion of the upper β-subunit leg. Scheme created with BioRender.com (**B**) 3D representation of aIIbB3 integrin PDB: 8T2V [65] with the different parts indicated.

As mentioned above, integrin structure is made up of an α-subunit and a β-subunit (Figure 3). In the α-subunit, the region located C-terminal to the β-propeller forms the “leg” of the α-subunit and includes three β-sandwich domains, along with a small genu (knee-like) domain [59,60]. The upper leg consists of the thigh and genu domains, while the lower leg is made up of the calf-1 and calf-2 domains. The β-subunit has a more complex structure: the β-subunit ‘inserted’ (I)-domain is embedded within the hybrid domain, which constitutes the upper part of the upper β-subunit leg. The remainder of the β-subunit leg is formed by a hybrid domain inserted into the plexin/semaphorin/integrin (PSI) domain, along with four epidermal growth factor (EGF)-like domains and a β-tail domain [61]. Ligand binding to integrins is facilitated by divalent cations, which are coordinated at metal ion-dependent adhesion sites (MIDAS) [56,59] (Figure 3A). This I-domain contains an interlinked linear array of three metal ion-binding sites, the central MIDAS flanked by the synergistic metal ion-binding site (SyMBS) and the so-called ‘adjacent to MIDAS site’ (ADMIDAS) [62].

Integrins are key cell receptors in a wide variety of processes, including cell adhesion, cell migration and cell–cell interaction, as well as acting as a physical and biochemical boundary between the cytoskeleton and ECM [63]. The regulation of these complex signaling processes is triggered by integrin-mediated recruitment of large multi-protein assemblies at the cell surface. These protein complexes are commonly referred to as integrin adhesion complexes (IACs) and mediate bidirectional signaling processes across the plasma membrane [64]. These processes are not limited to migration and include the regulation of gene expression, cell proliferation, cell survival, mitosis, cell polarity, differentiation and cross-talk with other receptor systems [57]. Recent studies have highlighted the importance of IACs during the cell migration process. Cells can adopt different migration modes and move as single cells or as collective sheets, dictated by cell-intrinsic features (for example, adhesion receptor expression, cell contractility and proteolytic activity) and cell-extrinsic parameters (such as ECM composition, stiffness and porosity) [63,64]. The chosen migration mode predominantly reflects the balance between actin-mediated cell protrusions, actomyosin contractility and adhesion to the ECM [56]. For example, the so-called amoeboid form of cell migration is associated with low adhesion to the ECM and high contractility, whereas mesenchymal migration requires strong integrin–ECM engagement and actin polymerization at the cell front. The ability of cells to switch migration modes is particularly relevant for cell migration during cancer progression, where transformed epithelial cells show alterations in the cytoskeleton and their IACs [64].

### 3.2. Integrin Activation

Integrins are expressed in the cell surface in an inactive state where they do not bind ligands and do not produce inside-out signals. This is a key feature for their biological function. For instance, platelet integrin αIIbβ3 is present at high density on blood-circulating platelets where it is inactive. On the contrary, platelets bind their major ligand, fibrinogen, form the plasma clot and aggregate, leading to thrombosis [58].

**Figure 4:**
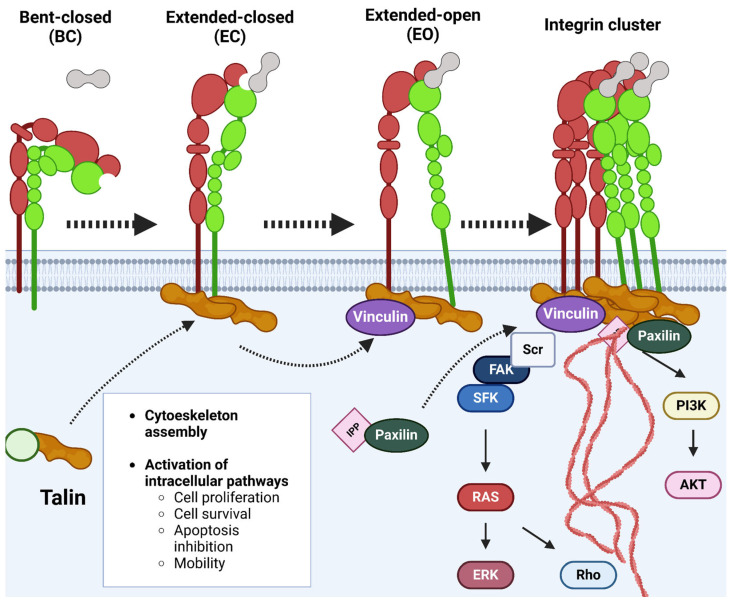
**Intracellular signaling triggered by integrin activation.** Depiction of sequential activation of integrins. Three conformational states have been described: Bent integrin-Closed headpiece (BC), the initial inactive state; Extended integrin-Closed headpiece (EC), when binding low-affinity ligands and Extended integrin; Open headpiece (EO) when binding high-affinity ligands. Closed headpiece conformations are stabilized by hydrophobic interactions between α- and β-subunits. Once activated, integrins form clusters that trigger intracellular signaling, including PI3K and FAK pathways. Created with BioRender.com.

Evidence from many studies using complementary approaches, such as cryo-electron microscopy [66] or fluorescence resonance energy transfer (FRET) [61], have established the prevailing concept according to which integrin heterodimers adopt three main conformational states (term closed, intermediate and open) with extensive global conformational rearrangements that enable integrin–ligand binding and activation (Figure 4). The initial inactive conformation, known as bent-closed (BC), describes integrins in a bent state with a closed headpiece, which become extended upon activation with either a closed (low ligand affinity) or open (high ligand affinity) headpiece [60,62]. Conformations with a closed headpiece are stabilized by interactions between the α-subunit and β-subunit, including hydrophobic interactions between the transmembrane helices and electrostatic contacts within the membrane-proximal helices of the cytoplasmic tails [67].

During integrin activation, the key feature is the physical separation between both α- and β-subunits (Figure 4). This event can be triggered by an extracellular ligand binding, regarding an “outside-in” activation, or intracellular signaling, promoting an “inside-out” activation [56]. These two different activations of the protein will be involved in different cellular responses. Inside-out signaling involves the recruitment of intracellular proteins, such as the large scaffolding protein talin, which connects the cytoplasmic tail of the integrin β-subunit to the actin cytoskeleton [68]. On the other hand, during outside-in signaling, high-affinity ECM ligands (such as FN) extend the extracellular domains and activate downstream signaling of several protein kinases, such as focal adhesion kinase (FAK) and the proto-oncogene tyrosine protein kinase Src [69,70].

Integrin activation is also controlled by divalent metal ions that bind the MIDAS site. The effect of these divalent ions has been proved in vitro, showing that Ca^2+^ has an important role in keeping integrins in an inactive state. The relative concentrations of Ca^2+^ and Mg^2+^ are critical as Mg^2+^ competes with Ca^2+^ at these sites, increasing the intrinsic ligand affinities and shifting the conformational equilibrium towards the high-affinity open state [62]. Furthermore, Mn^2+^ is a well-established super-activator of integrins [71], as well as proving its capacity to activate newly synthesized integrins upon their delivery to the plasma membrane and induce cell migration in cutaneous wounds in vivo [72].

### 3.3. Intracellular Events Triggered by Integrin Activation

As the major cellular receptors for the ECM, integrin family cell adhesion receptors are essential for cell migration. FAK, a non-receptor tyrosine kinase, plays an important role in signal transduction pathways that are initiated at sites of integrin-mediated cell adhesions [69]. The attachment of cells to certain ECM proteins, such as FN, collagen and laminin, leads to the clustering of integrins, followed by recruiting cytoplasmic proteins, such as the aforementioned kinase FAK or paxillin (Figure 4). During the FAK activation process, the β-cytoplasmic domain of activated integrins interacts with the FAK activator domain FERM [69]. This will induce a conformational change to allow for FAK auto-phosphorylation at Y397 and its exposure for binding Src family kinases, which phosphorylates additional sites on FAK, leading to its full activation [70]. Increased levels of FAK expression have also been correlated with the invasive metastatic potential of several human tumors or an augmented migratory capacity of endothelial cells (ECs) [70]. During the initial stages of cell spreading on FN when RhoA activity is low, focal complexes are formed that gradually increase in size and convert to focal contacts [73,74]. This early phase is efficiently stimulated by α5β3-mediated binding to ECM components, including FN. Subsequently, at later stages of cell spreading when the number of focal contacts containing α5β3 continues to increase randomly in the cell, focal contacts containing α5β1 disappear from the center while the new ones are formed at the tips of cell protrusions in mice fibroblasts. This latter process is accompanied by an increase in RhoA-mediated tension [73].

Furthermore, in cells, integrin activation is tightly regulated by its own inactivation, a process controlled by proteins that bind to the integrin cytoplasmic domain [75]. Many integrin-inactivating proteins, such as integrin subunit β1-binding protein 1 (ITGB1BP1, also known as ICAP1) and filamin, are β-tail-binding proteins that work by directly preventing the recruitment of talin and kindlin to integrins [76]. Additionally, the integrin inhibitor shank-associated RH domain-interacting protein (SHARPIN) has been found to regulate integrin activity and migration in cancer cells and white blood cells in vitro [77,78]. Initially, SHARPIN was shown to interact with α-integrin cytoplasmic tails through a conserved sequence shared by all integrin α-subunits [78]. More recent structural studies revealed that SHARPIN inhibits β1 integrin activation by associating with not only the α-subunit but also the β-subunit tail and kindlin 1, thus preventing talin recruitment [79].

Combined effects of soluble growth factors and integrins have been examined and are also well known. Cell adhesion acts as a trigger for a sort of intracellular pathway related with proliferation, such as the PI3K-AKT pathway or MAPK pathway [80]. Thus, in cells where growth factor receptor function is not affected by the ECM, the activation of these molecular signaling processes depends on cell adhesion and integrin activation [80]. However, the response to either cell adhesion or growth factors alone is quite low in most cases, while both stimuli together give a strong response. These results imply that integrins and growth factor receptors act upon different points in the pathway [81]. Since oncogenes are constitutively activated on normal cell growth processes, one can make predictions about the effects of oncogenes on cell growth based on the placement of the corresponding proto-oncogene within the integrin or growth factor receptor pathway. For instance, constitutive activation of a step on the integrin arm of the pathway before convergence should provide the affected cell with anchorage-independent and serum-dependent growth capacity [80].

In addition, integrin trafficking has recently come out as a key process that regulates integrin function in cancer cells. Understanding molecular mechanisms of this process is crucial to unveil tumor progression [82]. Thus, integrin heterodimers are continually internalized from the plasma membrane into endosomal compartments and subsequently recycled back to the cell surface. Evidence suggests that integrin trafficking regulates cell adhesion to the ECM, establishes and maintains cell polarity, redefines signaling pathways and controls migration [83]. The whole process is known to be regulated by members of the Ras-associated binding family of small GTPases, which function as molecular switches regulating vesicular transport [84,85]. Also, endocytosis mechanisms are related with clathrin and caveolin proteins [86]. Once internalized, integrins are mainly recycled back to the plasma membrane, although a portion of integrin α5β1 has been observed to move to lysosomes for degradation during migration [87].

## 4. Integrins in the Different Steps of the Metastatic Cascade

As it has been established, integrins play a crucial role in a wide range of pathological processes, as well as in development and tissue homeostasis. In consequence, its role on tumor development and metastasis is also well described [88]. During the metastatic cascade, tumor cells must migrate through different microenvironments and interact with a huge range of proteins and factors, in order to conduct the EMT and enter into the circulating blood [89]. Thus, in order to migrate and disseminate effectively across these diverse landscapes, cancer cells must adopt a state of plasticity that allows dynamic regulation of integrin expression, distribution and signaling outputs to support invasion.

In the development of a solid tumor, the mechanotransduction and signaling mechanisms described by integrins are altered. Therefore, tumor cells overexpress proliferation, survival and invasibility pathways [47,50,90]. Recent studies highlight integrins αvβ3 and α6β4 as positive regulators of tumorigenesis [90]. In the next sections, we will focus on the role of integrins in the different steps of the metastatic cascade.

### 4.1. Tumor Exosomes and Integrins for Pre-Metastatic Niche Formation

PMNs consist of a microenvironment that includes stromal cells, ECM, tumor-secreted exosomes, homing factors and immune cells that render the tissue for metastatic cell colonization [91]. In this context, integrins are involved in PMN formation because they are found indispensably in the membrane of EVs that prepare distant tissue. They allow exosomes to recognize and stop at the target tissue in the homing process and the promotion of the effects over the cells that receive the exosomes. They induce a phenotypic transformation through signal transduction. These integrins from EVs are similar to those contained in the cells of a primary tumor, since they come from their cytoplasmic membrane. Depending on the type of primary tumor, the integrin footprint in EVs can be representative: ITGαvβ6 is involved in prostate, breast, colorectal and lung cancer [92]; ITGβ1 is overexpressed in hepatocarcinoma, related to the activation of the β1-integrin-NF-κB signaling pathway in resident fibroblasts to convert them into CAFs [93]; ITGβ4 and ITGβ5 determine the tropism of EVs to the lung; specifically, ITGβ4 recognizes laminin in the lung parenchyma [11]. Once tumor exosomes reach distant tissue thanks to integrins, angiogenesis processes, regulation of the immune population and ECM remodeling are triggered [92].

### 4.2. Matrix Remodeling and Invasive Phenotype Acquisition

Force-mediated ECM–integrin interaction induces various downstream signaling events, known as mechanosignaling [94]. Repeated adhesion and de-adhesion of cells to the ECM substrate via integrins and the alteration of actin cytoskeletal reorganization mediate cellular processes such as cell migration [94,95]. This integrin-mediated signaling includes pro-survival and pro-apoptotic pathways, and their crosstalk with growth factor receptors mediates cellular signaling. As a result of the alterations in the ECM, tumors often are stiffer than normal tissue, which can be applied in clinical diagnosis [89,96]. The stiff matrix of the tumor leads to altered mechanosignaling. High ECM deposition and increased stiffness combined with integrin overexpression in various cancers trigger tumor promotion [89].

**Figure 5:**
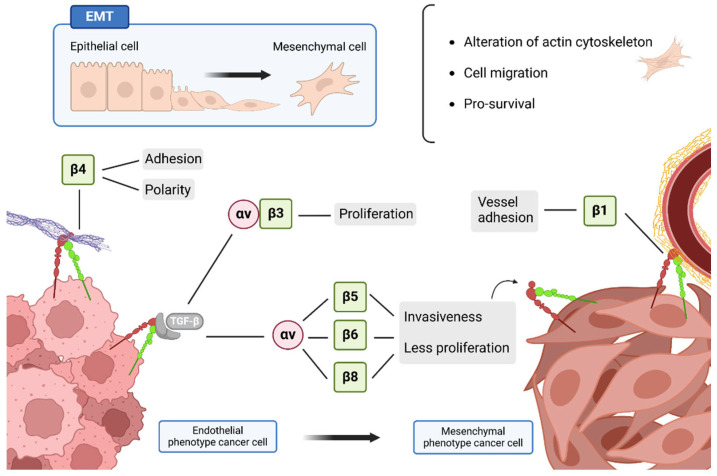
**Integrins involved in EMT.** The transition from an epithelial phenotype to a mesenchymal phenotype is essential to provide tumor cells with the migration and survival capabilities necessary to initiate the metastasis process. Many integrins are involved in EMT, since they trigger intracellular pathways by binding to cytokines and fibers of the ECM. When integrin β4 adheres to the matrix, it promotes adhesion and the loss of apico-basal polarity of cells with an epithelial phenotype. β1 integrin of mesenchymal cells is involved in adhesion to the BM of vessels to initiate the extravasation process. When TGF-β binds to integrin αvβ3, it drives cell proliferation, whereas if TGF-β binds to αvβ5/6/7, it promotes EMT by increasing invasiveness and decreasing proliferation. Overall, cells with a mesenchymal phenotype have an alteration in the regulation of cytokines for morphological change; migration is increased and apoptosis is inhibited. Created with Biorender.com.

The invasive phenotype in the cells of a primary tumor that allows the subsequent steps in the metastatic cascade is fundamentally due to EMT (Figure 5). Moreover, in addition to activating pro-migratory properties, integrins are essential for the activation of TGF-β, a key regulator of EMT [97]. To understand the role of integrins with TGF-β, it is necessary to know that first, it is synthesized in an inactive form. Initially, it forms a latent complex that is deposited in the ECM, surrounded by elastic fibrils and fibronectin. The cleavage of this latent complex into an active TGF-β is through proteases or by binding to integrins that recognize RGD sequences of fibronectin or a certain part of the latent complex. The active TGF-β can bind to its own receptors I and II, which triggers another type of signaling dependent or independent of Smad [98,99].

Different integrins participate in the EMT process (Figure 5). β1 integrins have been shown to induce the transcriptional program towards this invasive phenotype and tissue immunosuppression [50,90]. When overexpressed, β1 integrins also participate in tumor adhesion to the BM of blood vessels in brain metastases [100] and generate resistance to chemotherapies in hematological, lung and breast cancers [47,90,101,102]. Thus, integrin β1 is associated with aggressive tumor phenotypes prone to metastasize [47,90,101,102].

On the other hand, αvβ3 integrin recognizes TGF-β and activates the FAK/PI3K/AKT pathway, which promotes EMT and is associated with breast cancer cells with cisplatin resistance [103,104]. Activated TGF-β also binds to integrins αvβ5, αvβ6 and αvβ8, inducing tumor invasiveness and decreasing cell proliferation, promoting EMT and, therefore, metastasis [89,98,105]. Subsequently, genetic studies link integrin β4 with an alteration in cell adhesion and polarity, promoting invasion and hyperproliferation in breast tumors [106].

### 4.3. Intravasation, Dissemination and Extravasation

Tumor cells use β1 integrins to adhere to the BM of blood vessels in the primary neoplastic tissue [100]. Subsequently, intravasation is the process by which tumor cells cross the endothelium and reach the bloodstream or lymphatic system. It has been reported that a complex between c-Met tyrosine kinase receptor and β1 integrin promotes intravasation in breast cancer, initiating the metastatic dissemination phase [107].

Once they are in the bloodstream, CTCs form a metastatic embolus surrounded by platelets and FN deposited by the cells themselves. During dissemination, there is stimulation by integrins that bind to the different components of the embolus and trigger pro-survival pathways such as FAK/ERK, Rho and YAP [32]. One of the integrins involved is αvβ3, which protects CTCs from the physical stress of blood flow and immune recognition by NK cells [108,109]. Likewise, cytokines produced by CTCs, such as interleukin-8, attract neutrophils that surround the tumor embolus using β2 integrins [90].

When CTCs reach the target organ, they extravasate crossing the vascular endothelium, using the integrin αvβ3 to make their way through the endothelium, since pathways that modify the cytoskeleton are triggered and are helped by the secretion of MMPs [32,89]. Furthermore, β1 integrins show a high capacity to allow metastatic development in multiple organs and once they extravasate, and tumor cells can use them to adhere to pre-existing vessels and grow through vascular co-option (VCO) [109].

### 4.4. Awakening from Latency and Colonization of the New Tissue

When tumor cells reach the target tissue, they are in a latent state that has been initiated by the dissemination of CTCs. Therefore, the previous step to metastatic colonization is the exit from cellular latency, a phase that can extend over time in the form of micrometastases [33]. Integrins that measure signals between the ECM and metastatic cells intervene in cellular activation from a quiescent state to a proliferative and invasive state [90]. For example, in lung metastases from breast cancer, newly extravasated cells form a kind of filopodia, promoted by the adhesion of β1 integrins to the lung parenchyma and blood vessels, which activate FAK and initiate the exit from latency [89,109]. Another pathway initiated by this integrin for cellular activation is ILK/YAP in HER2+ breast carcinomas [32].

Colonization begins with cell proliferation and migration through the new tissue. In this process, the ECM and the TME are modulated; for this reason, the integrins expressed by these cells are essential, regardless of the metastatic destination [110]. Cell invasion is intrinsic to an increase in adhesion to matrix fibers and less intercellular adhesion [31]. The integrin αvβ1 of CAFs binds to TGF-β and FN and promotes an increase in ECM components [111,112]. Tumor cells migrate through it, guided by the binding to matrikines recognized by integrin β1 [113]. In addition, specifically, integrin α5β1 binds to FN, fibrinogen, fimbrin, osteopontin, thrombospondin and semaphorins, which helps colonization by initiating pro-migratory, pro-invasive and proliferative intracellular pathways [111,114,115]. Furthermore, it has been observed that in pancreatic cancer and melanoma, integrin subunits α6 and β3 are pro-tumorigenic, since they activate the ERK pathway by binding to activated K-RAS and B-RAF [116]. We also have to take into account that integrin adhesion can be modified in tumor cells, promoting invasiveness, as is the case of gain-of-function mutations in the *tp53* gene [117].

As we mentioned above, colonization and metastatic development also involve the reprogramming of the TME. In this case, there may be factors that promote the formation of blood or lymphatic vessels and that modulate the immune populations present. The integrin α6β4 has a pro-angiogenic effect, while the endothelial integrin α3β1 negatively regulates angiogenesis by decreasing the expression of vascular endothelial growth factor receptor 2 (VEGFR-2) in these cells, but in contrast, αvβ3 integrin crosslinking with this VEGFR-2 promotes angiogenesis [90,118]. Tumor integrins α3 and α6 mediate adhesion to tissue blood vessels [47]. Likewise, the integrin α4β1, which binds to FN and the counterreceptor VCAM1 (vascular cell adhesion molecule 1), is involved in lymphangiogenesis and lymph node colonization [119]. The development of blood vessels may involve the arrival of anti-inflammatory immune cells: inflammatory stimuli that activate PI3K promote the expression of the integrin α4β1 in tumor suppressor myeloid cells, which in turn allows the recruitment and extravasation of these cells [90]. In this case, anti-tumor CD8+ T cells migrate through the tumor by adhering to collagen fibers I and IV through the integrin α1β1 [120]. In contrast, tumors try to recruit and maintain pro-tumor cells, such as M2 macrophages that overexpress the integrin αvβ3 [121]. In lung metastasis of breast cancer, these macrophages express the integrin α4β1, which binds to the tumor counter receptor VCAM-1 and activates survival signaling in these oncogenic cells [122]. And finally, another aspect that it is important to know is about the interaction between the integrins and other cell receptors implicated in colonization development, such as α6β4 integrin, which crosslinks with some tyrosine kinase receptors involved in EGFR family members or α5β1 integrin, which can also interact with urokinase-type plasminogen activator receptor to promote proliferation, migration and survival of cancer cells [118].

In summary, the adhesion of tumor cells to the ECM involves numerous integrins that modulate mechanochemical signaling that is often altered, regulating matrix remodeling, invasiveness and survival involved in metastatic development and in the clinical resistance presented by this disease [47,50,90,111].

## 5. Integrins’ Role in Tumor Vessel Development

**Figure 6:**
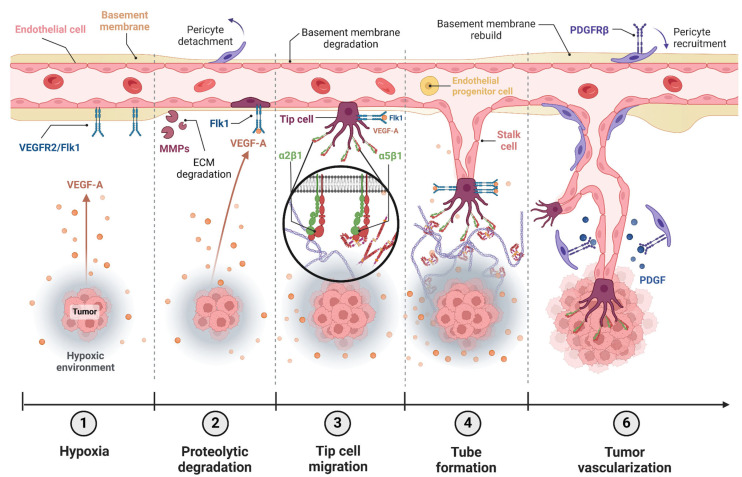
**Role of integrins in angiogenic sprouts.** (**1**) Sprouting processes begin with angiogenic stimuli such as hypoxia (HIF-α). Hypoxia can promote the production of vascular endothelial growth factor (VEGF). This molecule and its receptor VEGFR-2/Flk1 are the main actors of this process. (**2**) At the beginning of the sprouting process, basement membrane degradation occurs, together with the action of matrix metallo-proteinases and pericyte detachment. (**3**) Tip cell initiates migration through chemotaxis to the hypoxic area. Integrins are thought to play a key role in this process via mechanosignaling and matrix attachment. (**4**) The migration process continues. Stalk cells form the new blood vessel following the tip cell. (**5**) The new blood vessel is almost formed and acquires structural complexity. Platelet-derived growth factor (PDGF) stimulates the coverage of the new vessel by pericytes. Created with BioRender.com.

There are various mechanisms to form new blood vessels and remodel the vascular network [123,124,125,126,127], such as vasculogenesis, where vessels are formed de novo from precursor cells [127], or angiogenesis, which involves the formation of new blood vessels from pre-existing ones [126,127]. Except during embryonic stages, angiogenesis is the most important process at the physiological level, as well as in pathologies, being crucial in tumor development [125,127]. To trigger blood vessel growth, ECs must be exposed to a specific pro-angiogenic stimulus that “awakens” them, making them mobile, invasive and proliferative (characteristics expressed distinctly in various cellular phenotypes), leading to the formation of an endothelial sprout [123,124]. These stimuli can be injuries, hypoxia or growth factors induced by oncogenic cells [127,128] (Figure 6).

Tumor angiogenesis involves ECs activation, dissolution of the surrounding basement membrane, increased ECs proliferation and migration, tube formation, vessel anastomosis and pruning to form a vascular network. Among different physiological features involved in this process, integrins play a key role as adhesion molecules and physical signals transductors [129]. ECs express the following integrins: α5β3 and α5β5, the vitronectin receptors; α4β1 and α5β1, the FN receptors; α1β1 and α2β1, the collagen receptors α3β1, α6β1 and α6β4, the laminin receptors; and α9β1, the osteopontin receptor. Among all these, α5β3 was the first thought to be involved in pathological processes, as its expression was apparently increased in proliferating ECs [130,131]. Upregulation of integrins α5β3/α5β5 allows growing ECs to bind matrix proteins, including vitronectin, fibrinogen and FN. These adhesive interactions provide survival cues and traction for invading ECs and thus, promotes angiogenesis [132]. Then, α5β3 and α5β5 inhibitors, including low-weight antagonist molecules and antibodies, were developed and showed capacity to inhibit endothelial adhesion and reduce tumor neovascularization in a variety of in vivo models [133]. Furthermore, antagonists of β3 integrin are being used in clinical trials as anti-angiogenic therapy, including the humanized monoclonal antibody Vitaxin and the RGD mimetic Cilengitide [134]. Nevertheless, limitations arose when these molecules faced clinical trials and did not achieve successful angiogenesis inhibition [135]. However, β3 null and β3/β5 double-deficient mice produce vascular networks without obvious defects during developmental angiogenesis [136,137]. Taken together, these inhibition data suggest critical roles for αvβ3 and αvβ5 in angiogenesis, and highlight their importance as potential targets in anti-angiogenic therapy [138]. In addition with these inhibitor studies, mice lacking αv, β3 or β5 integrins exhibit extensive developmental angiogenesis. All αv-null mice have extensive sprouting angiogenesis and develop normally until embryonic day 9.5, and approximately 20% of them survive to birth [136]. β3-null mice are both viable and fertile, and developmental angiogenesis, including postnatal neovascularization of the retina, appears to be β3-independent [137]. β5-null mice are also viable and fertile and have no defects in wound healing, suggesting that adult angiogenesis is unaffected in these animals [139]. These results indicate that the precise role of αv integrins in angiogenesis is likely to be more complex than initially thought and raise the question of the importance of αvβ3 and αvβ5 integrins in adult pathological angiogenic processes [138]. Furthermore, VEGFR-2/Flk1, a key receptor molecule that plays an important role in angiogenesis and blood vessel development [140], has shown an increase in its expression in β3-null mice [138]. Flk1 expression is upregulated on new blood vessels [141], and antagonists that block the function of VEGF-A or Flk1 inhibit tumor growth and angiogenesis in mice [141,142]. Regarding these data, it is thought that this rise in Flk1 levels is responsible for angiogenesis boost in β3-null mice [143].

Driven by hypoxia, vascular endothelial growth factor (VEGF) is the main signaling element in angiogenesis [126,128]. ECs detect the chemotactic gradient of VEGF, following it to form the vascular sprout [124,126,127]. In this process, numerous cytokines and cellular factors are involved, such as angiopoietins; however, studies increasingly highlight the importance of the ECM proteins in the angiogenic process [126,144,145]. An excess of ECM rigidity provided by its components, such as collagen and FN, can impede angiogenesis. However, balanced stiffness facilitates adequate adhesion of ECs in the sprout for directed migration and proliferation; this adhesion is mediated by integrins that trigger intracellular signals involved in the angiogenic process [126].

Tumor cells attempt to regulate the ECM and secrete cytokines to meet their needs, manipulating the balance between matrix deposition and remodeling. High cellular compaction in neoplastic masses has been shown to induce the expression of type IV collagen, which constitutes the BM and LOX, which, in turn, seems to induce the release of VEGF [145]. Another protein secreted by oncogenic cells is CTHRC1 (collagen triple helix repeat-containing 1), which induces ERK and c-Jun N-terminal kinase phosphorylation in ECs, producing a pro-angiogenic effect supported by matrix remodeling through the promotion of MMP9 activity [146].

FN also contributes to angiogenesis by activating FAK, WNT/β-catenin and MAPK/ERK pathways, as this ECM component is the main ligand of α5β1 integrin. The upregulation of these pathways leads to the secretion of VEGF, CD31, Tie2 and Ve-cadherin and maintains an elevated signaling of hypoxia-inducible factor 1-alpha (HIF-1α) [147].

It is known that integrins have important functions during tumor vascularization and development, such as VEGF-dependent and VEGF-independent angiogenesis, by promoting ECs migration. Moreover, β1 integrin coordinates much broader functional activities such as inflammation, proliferation, adhesion and invasion; it has recently been implicated in therapeutic resistance in multiple solid cancer models [148]. β1 integrin-mediated resistance is thought to occur at the level of the tumor cells themselves. Then, integrin drives resistance to anti-angiogenic therapy by promoting multiple mechanisms at the interface of tumor cells and the microenvironment [149]. Distinct adhesion molecules expressed by cancer cells, including β1 integrin, α6 integrin and the axonal path-finding neural cell adhesion molecule L1 (L1CAM), have been shown to facilitate this attachment to vessels [150]. For specific examples, it has been probed that in a mouse model of brain-metastatic breast cancer, cancer cells adhered to brain blood vessels through either L1CAM or the cell adhesion receptor β1 integrin, and inhibition of these molecules blocked vessel co-option (VCO) and the formation of brain metastases [151]. After FN overexpression, a transformed phenotype in human fibrosarcoma cells has been shown. Consistently, it has been found that overexpressing FN receptor integrin α5β1 in Chinese hamster ovary (CHO) cells induces more peri-FN deposition, rendering CHO cells significantly less migratory and anchorage independent [152].

Conversely, anti-angiogenic properties are found where the fragmentation of ECM fibers releases peptides that inhibit blood vessel growth. For instance, the NC1(XIX) domain located at the C-terminus of the α1 chain of type XIX collagen associated with BM has been shown to inhibit angiogenesis, and the vasostatin-1 fragment of chromogranin A inhibits the effects of TNF-α and VEGF [153,154].

## 6. Therapies Based on Integrin Inhibition and Their Application in Metastasis

As we have seen in this review, integrins have a fundamental role in metastatic development, being intermediaries of communication between the ECM and intracellular signaling that determines the tumor phenotype and actions. The great variety of functions of integrins are spread into the different steps of the metastatic cascade. Therefore, it is worth highlighting, as current and future clinical perspectives, a series of therapies that address the functioning of integrins involved in cancer. The inhibition of these integrins can modify the ECM and the TME [155,156] making it favorable for the patient’s prognosis and reducing tumor survival, proliferation and mobility, as well as overcoming acquired resistance to other therapies.

Antibodies against these integrins and their molecular inhibitors have been synthesized [101,157]. Numerous current clinical trials have tried to determine their efficacy in different types of neoplastic pathologies (Table 1). However, these new integrin inhibitor drugs may prove to be very useful when combined with chemotherapy, radiotherapy and immunotherapy. Several studies have reported the therapeutic benefit of combining integrin β1 inhibitors with radiotherapy or 5-fluorouracil to overcome the resistance of certain cancers to these treatments [90,102,158,159].

Apart from the integrin inhibitors, some inhibitors of related molecules, such as FAK or Src, have completed clinical trials (NCT00563290; NCT00835679, NCT00546104, NCT00597038, NCT01306942, NCT04161391, NCT00528645) and their results have been posted, especially those using Dasatinib, a Src inhibitor. However, very few clinical trials have published their results. With respect to integrin inhibitors, it is very important to remark that Abituzumab (EMD525797), humanized monoclonal antibody against integrin αv heterodimers, has been tested in combination with cetuximab and irinotecan in patients with metastatic CRC. In this study, doses up to 100 mg were well tolerated. A trend to improve survival was observed at 500 mg or 1000 mg. Moreover, patients with high levels of αvβ6 integrin showed poor prognosis [160]. This study is especially relevant in those patients with poor prognosis. As we can see from clinical trials, it is very important to know and to be able to detect the levels of the different integrins to predict prognosis and also response to the different integrin inhibitors. The clinical trials are showing that further studies, especially in combination with lower doses of chemotherapies, are needed in the future.

## 7. Conclusions

Despite the extensive studies that have addressed the identification, characterization and function of integrins, which have led to numerous clinical trials (see Table 1), many challenges remain in the study of these proteins and their potential therapeutic use in cancer treatment.

Addressing the TME and its changes throughout tumor progression, as well as following various chemotherapy, radiotherapy or immunotherapy treatments, requires understanding the alterations in the expression or localization of integrins, as well as the different compositions of the ECM. For the development of integrin inhibition-based therapies, unveiling their role in both the primary tumor and metastasis will be very important. Moreover, it is also important to study pharmacokinetics with precision because the plasma concentrations of integrins inhibitors, such as RGD mimetics, can vary during the first hours post-administration, and may promote opposite effects [161].

As we have seen, many integrins can be expressed in different cell types within a tumor and its microenvironment. For this reason, understanding the components of the TME will be essential to determine the different treatments that will be combined with integrin inhibitors. Furthermore, TME changes induced by cancer therapy is a challenge for understanding the pathophysiology and overcoming resistances. These treatments alter the tumor immune response, as well as the composition of the ECM and tumor vascularization. For this reason, therapies designed with integrin inhibitors may face these limitations.

In conclusion, it is particularly interesting to explore the utility of molecules that bind to different integrins and hold significant diagnostic potential. All of this could contribute to advancing personalized medicine based on integrins.

## Figures and Tables

**Table 1:** Clinical trials affecting integrin functionality in metastasis.

Clinical Trial Code	Molecule	Target	Pathology	Status
NCT06389123	[68Ga] Ga DOTA-5G [177Lu] Lu DOTA-ABM-5G	αvβ6 integrin	Metastatic Cancer	Recruiting
NCT00066196	MEDI-522	αvβ3 integrin	Metastatic Melanoma	Completed
NCT06228482	[68Ga] Ga DOTA-5G	αvβ6 integrin	Metastatic Non-Small-Cell Lung Cancer (NSCLC)	Recruiting
NCT00100685	Volociximab	α5β1 integrin	Metastatic Renal Cell Carcinoma (RCC)	Terminated
NCT00099970	Volociximab	α5β1 integrin	Metastatic Melanoma Not Previously Treated With Chemotherapy	Completed
NCT01664273	Plasmid AMEP	α5β1 and αvβ3 integrins	Disseminated Cancer	Terminated
NCT03164486	18F-αvβ6-Binding-Peptide	αvβ6 integrin	Breast, Colorectal, Lung or Pancreatic	Active, not recruiting
NCT03688230	Abituzumab	ανβ6 integrin	Metastatic Colorectal Cancer	Withdrawn
NCT06435741	99mTc-3PRGD2	αvβ3 integrin	Advanced Gastric Cancer	Not yet recruiting
NCT00915278	PF-04605412	α5β1 integrin	Solid tumors	Terminated
NCT01008475	EMD 525797	α integrins	Subjects With K-ras Wild-Type Metastatic Colorectal	Completed
NCT05101655	NA	NA	Lung Metastasis of Osteosarcoma	Completed
NCT04712721	68Ga-FF58	αvβ3 and αvβ5 integrin	Solid tumors	Terminated
NCT01806675	18F-FPPRGD2	Imaging of αvβ3 integrins	Glioblastoma Multiforme (GBM), Gynecological Cancers and Renal Cell Carcinoma (RCC)	Completed
NCT06460298	ProAgio (Anti-vβ3 Integrin Cytotoxin)	αvβ3 integrin	Metastatic Triple-Negative Breast Cancer	Recruiting
NCT00705016	Cilengitide	αvβ3 integrin	Squamous Cell Carcinoma of the Head and Neck (SCCHN)	Terminated
NCT04665947	[68Ga] Ga DOTA-5G and [177Lu] Lu DOTA-ABM-5G	αvβ6 integrin	Pancreatic cancer	Recruiting
NCT00401570	Volociximab	α5β1 integrin	Metastatic Pancreatic Cancer	Completed
NCT00537381	CNTO 95	αv integrins	Metastatic Hormone Refractory Prostate Cancer	Completed
NCT01961583	[18F] Fluciclatide	αvβ3 and αvβ5 integrins	Metastatic Renal Cell Carcinoma	Terminated
NCT00072930	MEDI-522	αvβ3 integrin	Metastatic Androgen- Independent Prostate Cancer	Completed
NCT04152018	PF-06940434	αvβ8 integrin antagonist	Advanced or Metastatic Solid Tumors	Active, not recruiting
NCT00684996	MEDI-522	αvβ3 integrin	Unresectable or Metastatic Kidney Cancer	Terminated
NCT01327313	EMD525797	αv integrins	Solid tumors	Completed
NCT01849744	VS-4718	FAK	Metastatic Non-Hematologic Malignancies	Terminated
NCT00563290	Dasatinib	Src	Metastatic Squamous Cell Skin	Completed
NCT00835679	Dasatinib	Src	Colorectal Cancer Patients With Resectable Liver Metastases	Terminated
NCT00546104	Dasatinib	Src	Advanced Breast Cancer	Completed
NCT01335269	BI 853520	FAK	Metastatic Non-hematologic Malignancies	Completed
NCT00597038	Dasatinib	Src	Melanoma	Completed
NCT01306942	Dasatinib	Src	Metastatic breast cancer	Completed
NCT04161391	TPX-0046	Src	Solid tumors	Completed
NCT00528645	AZD0530	Src	Small-cell lung cancer	Completed
NCT03993873	TPX-0022	Src	Advanced NSCLC, Gastric Cancer or Solid Tumors	Active, not recruiting
NCT01015222	Dasatinib	Src	Advanced cancers	Completed
NCT00504153	Dasatinib	Src	Metastatic Colorectal Cancer	Completed
NCT00277303	XL999	Src	Metastatic Colorectal Cancer	Terminated
NCT00277316	XL999	Src	Metastatic Renal Cell Carcinoma	Terminated
NCT01505413	Erlotinib	Src	Advanced Pancreatic Cancer	Completed
